# Microscopic Insights into a Ligand Escape Pathway and Energetics in Leucine–Isoleucine–Valine Binding Protein

**DOI:** 10.1021/acsomega.5c12025

**Published:** 2026-04-08

**Authors:** Jayita Das

**Affiliations:** Department of Chemistry, Boston University, Boston, Massachusetts 02215, United States

## Abstract

Periplasmic binding proteins (PBPs) are a large family of receptors and transporters present in Gram-negative bacteria, which play a pivotal role in cellular transport. PBPs have a distinct two-domain architecture that undergoes large conformational transitions through hinge motion. In particular, leucine–isoleucine–valine binding protein (LIVBP) can sense specific amino acid side chains and undergoes a transition from an open conformation to a closed conformation, which is traditionally viewed as a ligand-induced conformational change. Although many studies focused on conformational fluctuations of LIVBP, the same attention was not paid to the microscopic and energetic aspects of ligand escape. Here, μs long atomistic molecular dynamics and well-tempered metadynamics simulations are used to understand the role of the ligand (namely, isoleucine) in the conformational transition/selection of the LIVBP. Furthermore, the pathway, energetics, and sequence of events during ligand escape/unbinding are unveiled from a microscopic perspective. The ligand escape is a two-step process in which the domain separation precedes the ligand escape. However, the reverse is also observed. Water is found to play an important role in the ligand unbinding process as well as in providing stability to the closed conformation, in the absence of the ligand, by forming bridging hydrogen bonds between two domains.

## Graphical abstract

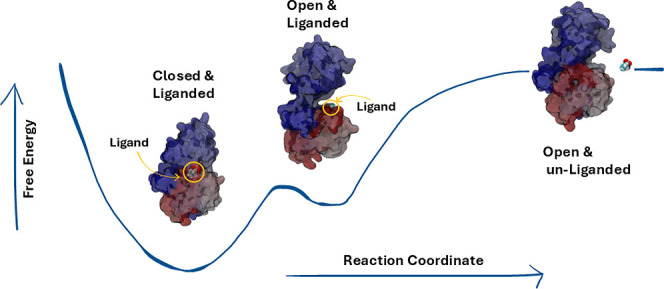

## Introduction

Bacteria sense small molecules and transport them into the cytoplasm with the help of periplasmic binding proteins (PBPs).^1–3^ These nonenzymatic receptors are present in Gram-negative bacteria (such as *Escherichia coli*, *Pseudomonas aeruginosa*, *Yersinia pestis*, etc.) which exhibit dramatic conformational transitions. PBPs play important roles in various cellular processes such as uptake and transport of metabolically important solutes (amino acids, sugars, and ions), chemotaxis, and quorum sensing. They are also an integral component of the ATP-binding cassette transport systems. PBPs have a high affinity for their substrates, which is conferred by a set of hydrogen bonds that interact with the substrate.^4^ PBPs can also fulfill a biosensing niche for nonimmunogenic targets.^1,5^

PBPs typically consist of two distinct domains connected by a hinge region. The ligand binding site is located in a cleft between the two domains.^4,6^ Binding of the ligand induces a conformational change. This is commonly referred to as the “Venus flytrap” model of binding.^7^ Such conformational changes are crucial to their function. The Leucine–Isoleucine–Valine (LIV) binding protein is a PBP involved in the transport of leucine, isoleucine, and valine, which are essential branched-chain amino acids. The ability to import branched-chain amino acids is critical for bacterial growth and survival, especially in nutrient-limited environments.^8–11^ The structure of LIV-binding protein was elucidated using high-resolution X-ray crystallography, representing the protein in its superopen form (PDB ID: 1Z15) and ligand-bound forms (PDB IDs: 1Z16, 1Z17, and 1Z18).^4^

Trakhanov et al. reported the ligand-free and ligand-bound structures of LIV-binding protein accompanied by normal-mode analyses and short targeted molecular dynamics (TMD) simulations.^4^ The root mean squared deviation of the open and closed structures is approximately 6.5 Å.^12^ It was identified that the ligand is attached to the protein cleft with the help of several hydrogen bonds (HBs) and van der Waals (vdW) interactions. Residues Ser-79, Thr-102, and Ala-100 from domain-2 and Tyr-202 and Glu-226 from domain-1 form multiple hydrogen bonding interactions with the ligands.^4^ Additionally, the ligand could get enthalpic stabilization through vdW interactions with neighboring residues, namely, Tyr-18, Leu-77, Leu-78, Tyr-150, and Phe-276.^4^ As the three ligand-bound structures are almost identical, the isoleucine-bound structure is chosen in this study as a prototype of the closed form.

Studies involving conformational transitions of various proteins have been on the forefront of theoretical and experimental biophysics for several years.^13–19^ Although a detailed simulation study on the LIV-binding protein is missing in the literature, similar PBPs were explored. Tang et al. used paramagnetic NMR on ‘unliganded’ maltose binding protein (MBP) to discover that there exists a rapid exchange (in the ns to μs regime) between the open (∼95% population) and semiclosed (∼5% population) forms.^14^ McCammon and colleagues investigated MBP by using accelerated MD simulations and continuum electrostatics calculations.^13^ They reported the existence of a semiclosed conformation which is stabilized due to hydrophobic interactions. Consistent with earlier experimental results by Tang et al.,^14^ the open form was found to be slightly more stable than the closed form but with roughly the same total energy. Based on the simulations, they hypothesized a two-step mechanism for the open → close state. The first step is the formation of a semiclosed state followed by a fast “ligand induced fit” mechanism. Salopek-Sondi et al. investigated the role of residue number 18 in two PBPs, namely, leucine-binding protein and LIV-bonding protein. Interestingly, they found the presence of Trp-18 disallows valine and isoleucine in the case of leucine-binding protein.^20^ In LIV-binding protein, the same position is occupied by Tyr which allows all three amino acids.

Despite the earlier works, several important aspects have remained poorly understood, especially for the LIV-binding protein. In this paper, the following questions are addressed: (i) What are the energetics, pathway(s), and sequence of events of ligand unbinding/escape? (ii) Can the unliganded closed form stabilize? If so, what could be the microscopic origin? In this paper, the questions are answered with the help of both unbiased and biased atomistic MD simulations. As water is known to play an important role in such processes,^21,22^ the present study also aims to provide insights into the role of water in modulating conformational stability and ligand escape in PBPs.

## Methods

The initial atomic coordinates were obtained from the protein data bank (PDB ID 1Z17) for the ligand (isoleucine, Ile) bound form.^4^ The files required to run MD simulation with GROMACS^23^ (version 2021.3) were obtained from the CHARMM-GUI server.^24^ In addition to two microsecond long unbiased simulations, five independent one-variable and two-variable metadynamics simulations were carried out to estimate the free-energy barrier of ligand escape and the sequence of events during the process.

The protein was placed inside a (10 nm)^3^ cubic box filled with TIP3P water and 0.15 M Na^+^ and Cl^–^ ions. The box size is sufficient to avoid periodic image interaction. Periodic boundary conditions were imposed in all three directions. The systems were first energy minimized, followed by a 10 ns equilibration. Then two independent unbiased production runs were performed. All the simulations used the CHARMM36m force field in NpT ensemble (*p* = 1 bar and *T* = 300 K) with the modified TIP3P water model.^25^ To fix the average temperature at around 300 K, the V-rescale thermostat^26^ was used (τ_T_ = 1.0 ps), and to keep the average pressure around 1 bar, the C-rescale barostat^27^ was used (τ_P_ = 5.0 ps and compressibility 4.5 × 10^–5^ bar^–1^). The leapfrog algorithm was employed to integrate Newton’s equations of motion with a time step (d*t*) of 1 fs (for equilibration) and 2 fs (for production run). The coordinates were saved in every 10 ps. The Verlet cutoff scheme was used. For vdW and short-range electrostatic interaction, a 1.2 nm cutoff was used. For long-range electrostatic interactions, the Particle Mesh Ewald (PME) algorithm^28^ was used with an FFT grid spacing of 0.12 nm. All bonded interactions were constrained using the LINCS algorithm.^29^

To calculate the free-energy barrier of the ligand (here, Ile) escape from the binding site, well-tempered metadynamics simulations^30^ were performed using PLUMED^31^ (patched with GROMACS). The collective variable was chosen as the distance between the *C*_α_ of Glu-328 and the center-of-mass (COM) of the ligand. The *C*_α_ of Glu-328 resides at the junction of the two domains and acts as a fulcrum to the hinge motion (red bead in Figure 1B). Five independent one-variable metadynamics runs were performed to check the robustness. The hill deposition frequency was set to 500 steps (=1 ps) with a bias-factor of 5. An upper wall was placed at *d* = 5 nm, to prevent sampling of uninteresting regions. All other simulation parameters and protocols were the same as those in the previously discussed unbiased MD setup. The metadynamics trajectories were truncated up to the time point when the ligand left the binding cleft.

**Figure 1:**
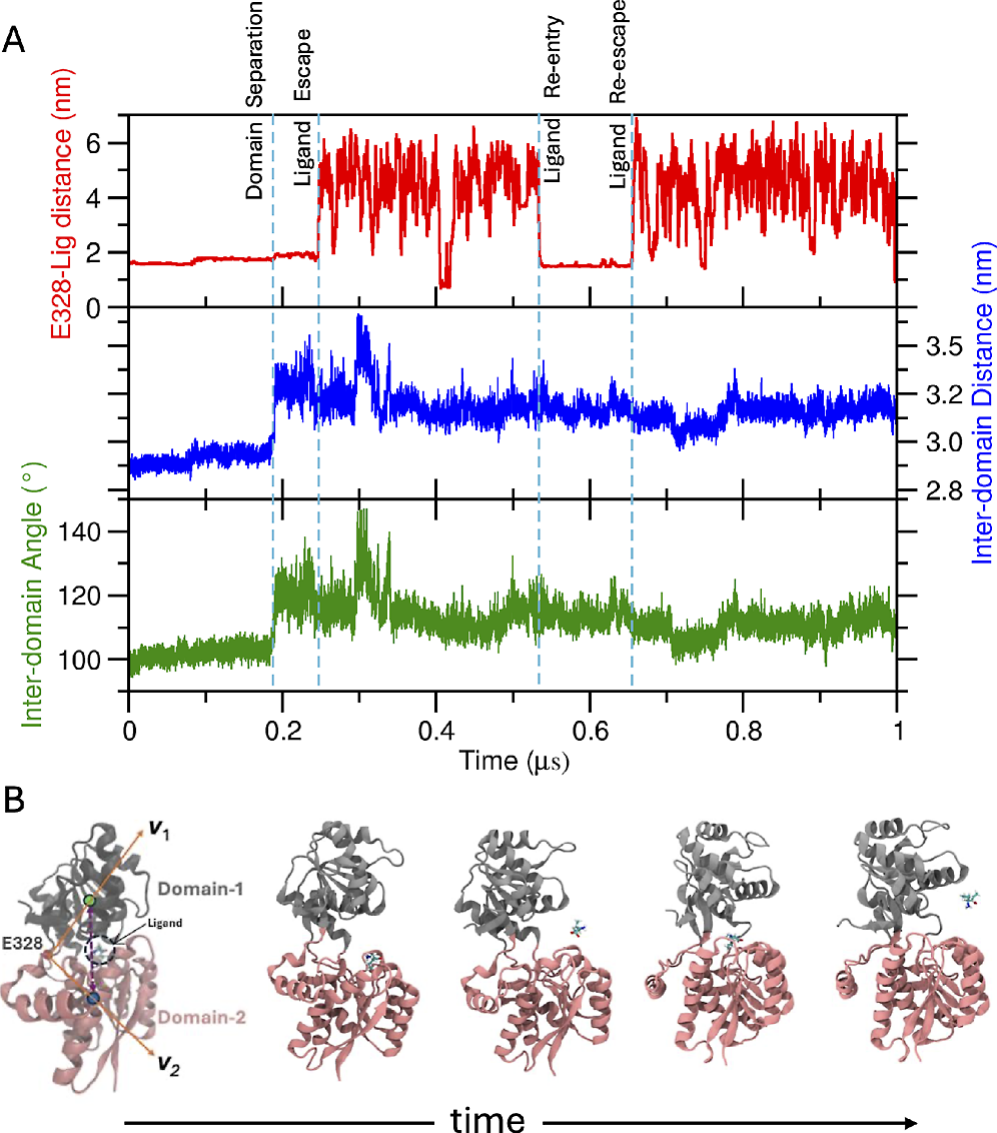
Observations from one of the two unbiased MD simulation of the ligand-bound closed form, ‘trajectory-1’: (A) The top panel (red trace) shows the protein–ligand distance (measured between E328 *C*_α_ and ligand COM) with time and the bottom panels (blue and green traces) show the change of the interdomain distance and angle, respectively, with time. (B) Representative snapshots from the trajectory showing the sequence of events marked in panel ‘A’ with vertical dashed lines.

To understand the synergy between the domain opening and ligand escape processes, a two-variable well-tempered metadynamics was carried out with two collective variables, namely, (a) the distance between the *C*_α_ of Glu-328 and the center-of-mass (COM) of the ligand and (b) the interdomain COM distance. The well-tempered metadynamics was run for 120 ns to obtain a converged free-energy landscape (FEL). The convergence is ensured by plotting the one- and two-dimensional FEL at different end points, ensuring the diffusive behavior of *d*_P–L_, and by plotting Δ*G*_binding_ with time (Supporting Information, Figure S1). The Gaussian hill height (at *t* = 0) was chosen to be 0.5 kJ/mol and the hill widths were fixed at a small value of 0.02 nm to explore the sharp features of the FEL. The analyses were preformed using the GROMACS postprocessing tools and sometimes using Python scripts. The visualizations were preformed using the Visual Molecular Dynamics (VMD)^32^ software and graphs were plotted using Python.^33^

## Results and Discussion

Conformational fluctuations and flexibility of a protein are key ingredients to their function, ligand binding, oligomer formation, and aggregation.^34–36^ The two globular domains, termed domain-1 (residues 121–248 and 329–344) and domain-2 (residues 1–120 and 249–328) in this study (Figure 1B), form a deep binding cleft where the ligand is accommodated. Upon ligand binding, the protein undergoes a significant conformational change, transitioning from an open to a closed state. From the X-ray crystallography structures, the interdomain center of mass (COM) distance for the superopen form is ∼3.4⁡nm and that for the ligand-bound closed form is ∼2.77⁡nm . In the absence of a ligand, LIV-binding protein was found to predominantly exist in an open conformation,^14^ with the two lobes positioned apart, allowing for rapid scanning and binding of branched-chain amino acids. The ligand (Ile) is held at the cleft region between two domains by nonbonded interactions which include electrostatic and hydrophobic attractions. The former appears in the form of hydrogen bonding that plays an important role in providing the much needed enthalpic stabilization. As discussed in the introduction, Ser-79, Thr-102, Ala-100, Tyr-202, and Glu-226 form multiple HBs with the ligands.

Ligand (and small molecule) binding/unbinding to proteins is a topic of great interest both from biophysical and medicinal perspectives.^37–40^ From the 1 μs unbiased MD simulation trajectories of the ligand-bound protein, the distance between C_α_ (*E* 328) and ligand-COM was calculated, as well as the interdomain separation distance and interdomain angle. Figure 1A shows the data for ‘trajectory-1’. The protein maintained its interdomain distance around 2.85 nm in the beginning of the trajectory which is very close to the value of 2.77 nm, as observed from X-ray crystallography data.^4^ At around 200 ns, the interdomain distance and angle showed a sharp increase, indicating domain separation. After that, the protein fluctuated between its superopen conformation (interdomain distance ∼3.4 nm) and open conformation (interdomain distance ∼3.0 nm). In this particular trajectory, the domain separation happened ∼40 ns before the ligand escape, as marked in Figure 1A. During that 40 ns, the ligand was found to be attached to domain-1 (Figure 1B). Interestingly, the ligand rebound to domain-1 around 500 ns. However, the ligand rebinding could not induce the “Venus flytrap”-like motion to close the two domains. The protein continued to remain in the open state and the ligand re-escaped after another ∼100 ns. A similar observation from ‘trajectory-2’ is provided in the Supporting Information (Figure S4), although ligand re-entry was not observed there.

A microscopic analysis (Figure 2A) revealed an interesting sequence of events at the ligand binding pocket. At some arbitrary *t* = 0, the ligand remained H-bonded to the four polar (and charged) amino acids: Y202 and E226 from domain-1 and S79 and T102 from domain-2 (frame *t* = 0 in Figure 2B). At about 100 ns, the H-bond between Y202 and the ligand was disrupted (state-1 in Figure 2D). After the domain separation at ∼200 ns, the ligand maintained its H-bonded interactions with S79 and T102 from domain-2, but not with Y202, and E226 from domain-1 (state-2 in Figure 2D). When the ligand re-entered the cleft region, it established H-bonded interaction with S79, T102, and E226 (state-3 in Figure 2D). State-3 is similar to state-1, as can be seen from the magnified snapshots: the –COO^–^ of the ligand formed H-bonds with S79 and T102, and the –NH_3_^+^ of the ligand forms H-bonds with E226. These results suggest that the ligand preferentially interacts with the domain-2 cleft side chains. Although it is quite remarkable to observe such ligand unbinding and rebinding from an unbiased all-atom trajectory, in order to extract the information on the energetics of the process, one needs the help of enhanced sampling techniques as discussed below.

**Figure 2:**
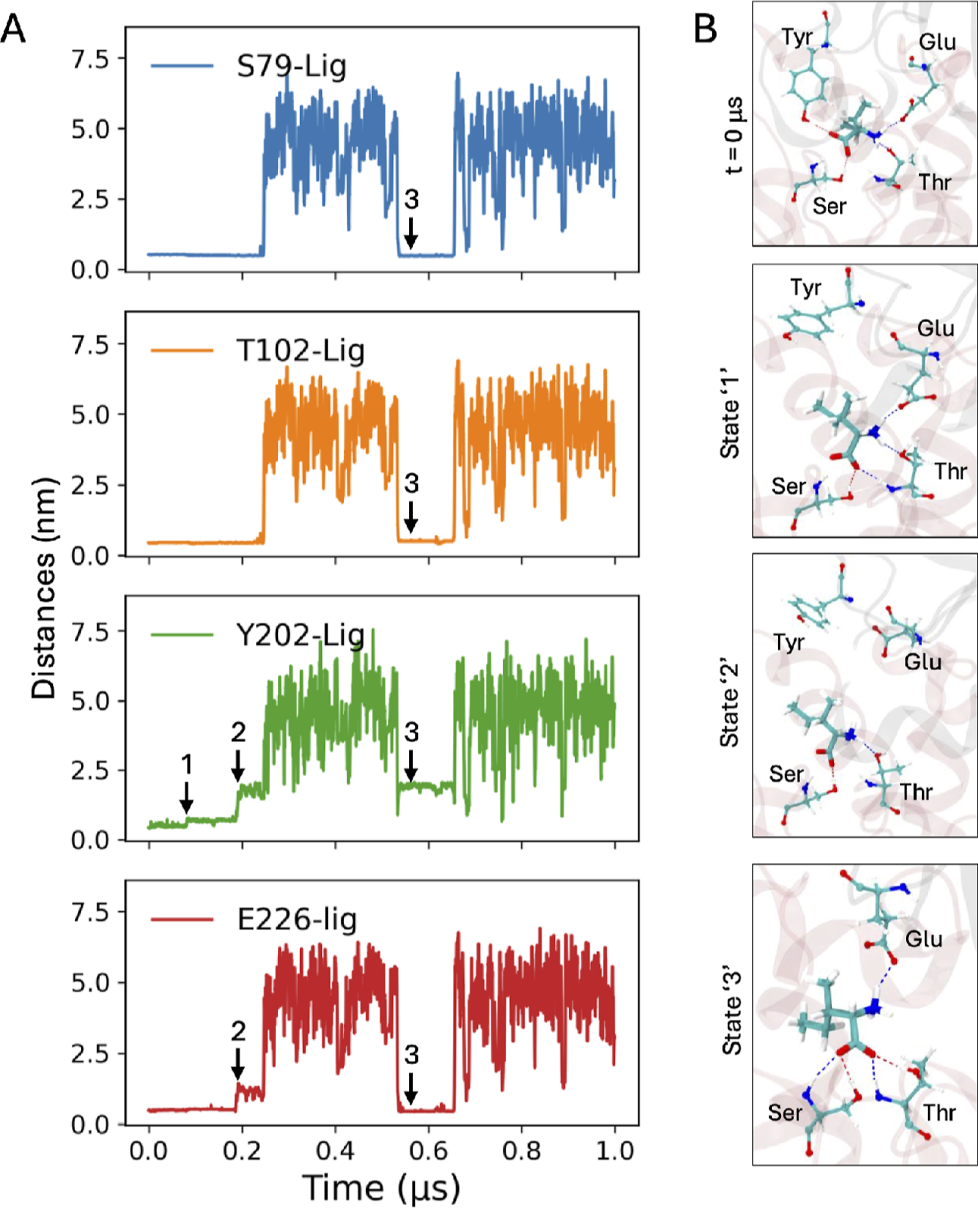
Microscopic dynamics of ligand escape at the cleft region: (A) Distance of the COM of the ligand from the cleft amino acids, namely, S79, T102, Y202, and E226. (B) Snapshots of magnified views of the cleft region showing the different states marked in panel ‘C’.

First, the results from the one-variable metadynamics are detailed. Here, one (slower) collective variable (distance between Glu-328 *C*_α_ and COM of the ligand) was biased and the other (faster) collective variables (such as water coordination, domain movement, etc.) were kept unbiased. From a metadynamics simulation, the dynamics of the ‘faster’ collective variables can be interpreted while biasing a­(the) ‘slow’ variable(s), primarily due to the separation in time scales of those processes.^30,41,42^

Based on the five independent metadynamics trajectories, two distinct escape pathways/mechanisms were observed. A pictorial depiction of the two paths is shown in Figure 3A with the help of snapshots. In Figure S2 (Supporting Information), path-I and path-II are shown with the help of two variables: (i) the distances between the ligand and the fulcrum of the protein (*C*_α_ of Glu-328) which is also the collective variable for metadynamics along which the system is biased and (ii) the interdomain separation distance which is unbiased. In one pathway (path-I), the domain separation occurred before the ligand escape. In another pathway (path-II), the ligand escape either occurred without the domain opening or it happened synchronously. In a previous targeted-molecular-dynamics study, Trakhanov et al. studied ligand unbinding from LIV-binding protein and found the domain-2 bound state (i.e., Ile H-bonded to S79 and T102) before the unbinding happened.^4^ However, the study did not explore the alternative pathway or provide estimates of the free-energy barrier for ligand escape.

**Figure 3:**
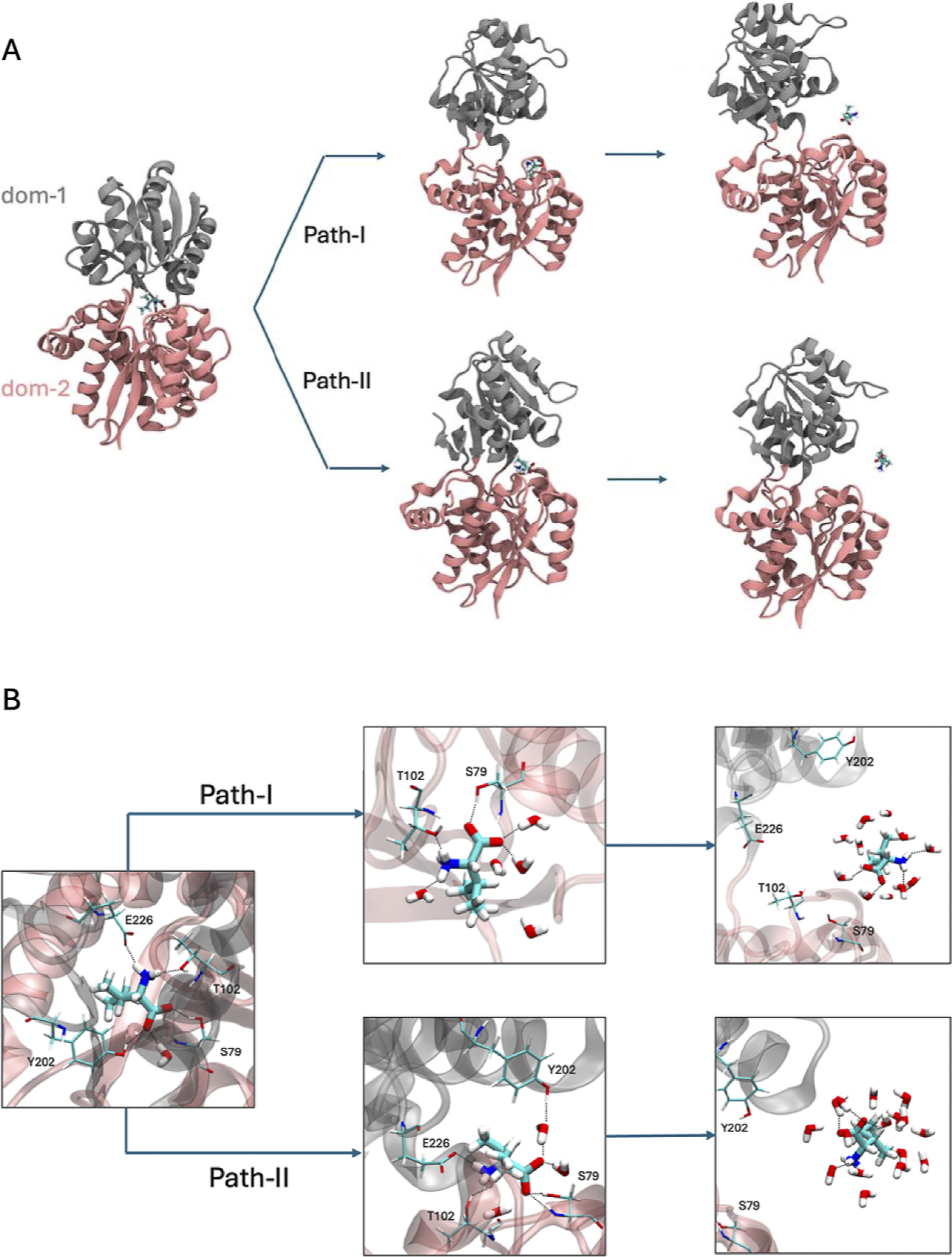
(A) Pathways I and II of ligand (Ile) escape from the cleft of LIV-binding protein. In path-I, the domain opening occurs first, then the ligand escapes. In path-II, the domains slightly open up, but eventually, the full domain opening happens simultaneously with ligand escape. (B) Magnified views of the ligand and its surroundings for the two ligand escape pathways shown in ‘A’. Initially, the ligand (Ile) was H-bonded to four amino acid side chains: S79, T102, Y202, and E226. Along path-I, the ligand remained attached to domain-2 through H-bonding interactions with S79 and T102 and partially solvated by a few water molecules, followed by the full escape of the ligand solvated by water molecules. In the intermediate of path-II, the ligand retained H-bonding interaction with three out of the four side chains: S79, T102, and E226. In addition, a water-mediated bridging H-bond is formed between the ligand and Y202.

In Figure 3D, the roles of water molecules in stabilizing the intermediate and the ligand-unbound states are shown. In path-I, the intermediate is the open form where the ligand is attached only to domain-2 (also observed from the unbiased trajectory). In that state, water molecules partially solvated the ligand as well as the polar side chains of domain-1. Subsequently, when the ligand fully escaped the binding pocket, water molecules completely solvated the ligand by forming several hydrogen bonds. In the second pathway, the intermediate was not a fully open conformation therefore the ligand retained majority of its H-bonding interactions with both the domains. Here, a few water molecules formed a stable hydrogen bonding interaction with the ligand and also bridging bonds, as shown in Figure 3D. Similar to pathway-I, the ligand escape was facilitated by a complete hydration of the ligand.

In Figure 4, the results and observations from two-variable well-tempered metadynamics are shown. The two-dimensional free-energy landscape (2d-FEL) showed several deep and shallow minima (Figure 4A). A one-dimensional projection of the 2d-FEL is shown with respect to the protein–ligand distance in Figure 4B. The states are marked with numerals, and the representative snapshots are shown in Figure 4C. ‘State-1’ is the native state where the protein was in its closed conformation and the ligand was accommodated inside the ligand binding cleft. The minimum energy pathway, as observed from the 2d-FEL, took the system from its ‘state-1’ to ‘state-2’, which is an open conformation of the protein with the ligand attached to its domain-2 (also observed from the unbiased trajectory). The transition from ‘state-1’ to ‘state-2’ was termed as pathway-I, described in Figure 3A, where domain separation occurs before ligand escape. There is a shallow metastable minima denoted by ‘state-3′ (in Figure 4A) where the ligand is not in the cleft region but resides at the periphery of domain-2 near residues D32, K36, I261, and Y281 (snapshot in Figure 4C). ‘State-4’ is the ligand unbound state where the protein preferably adapted to an open conformation. Another well ‘state-5′ denotes the ligand unbound closed conformation of the protein (a detailed understanding of this state is provided later). The transition between states ‘4’ and ‘5′ transforms the protein from an open to a closed conformation but without the ligand. On the other hand, the transition between states ‘1’ and ‘2’ was the domain open → closed transition when the ligand was present at the binding cleft.

**Figure 4:**
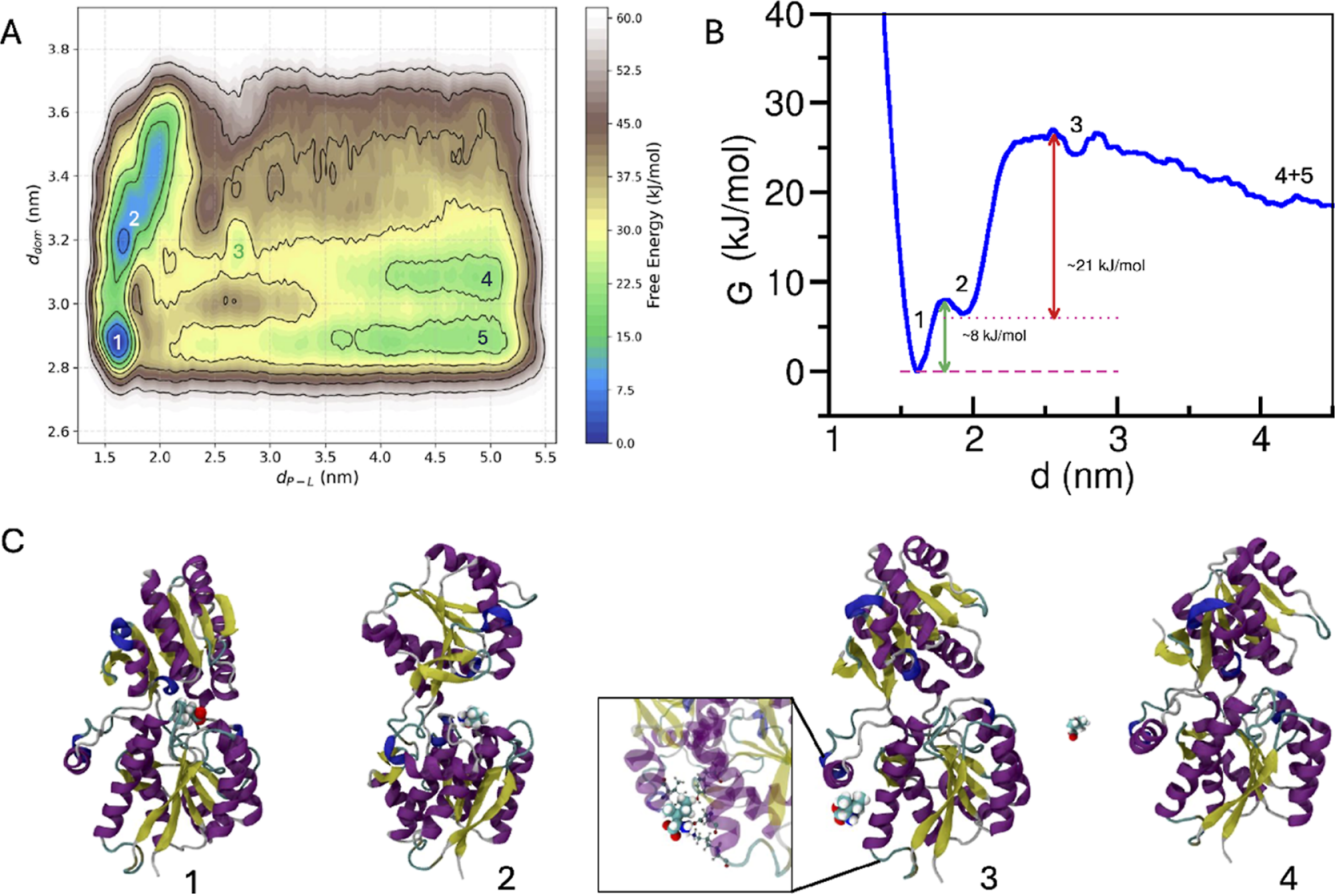
Results from two variable metadynamics: (A) two-dimensional free-energy landscape (2d-FEL) with respect to two collective variables, namely, the interdomain distance (*d*_dom_) and the protein ligand distance (*d*_P–L_). (B) The projection of the 2d-FEL on the protein–ligand distance axis. (C) Representative snapshots of the four states as identified from the different wells shown in panel ‘A’ and marked with numerals.

The general consensus for PBPs is that the ligand shifts the conformational ensemble to the closed form and without ligand, the protein stays as the open/superopen/semiopen form.^13,14^ To further investigate this, three separate 100 ns unbiased all-atom trajectories were produced, corresponding to the open, closed (liganded), and closed (unliganded) systems. From Figure S3 (Supporting Information), it can be seen that the closed (unliganded) form can survive without the ligand acting as an anchor between the two domains. From these shorter unbiased simulations, important information can be extracted regarding the role of water in the stability of the closed form of the LIV-binding protein without any ligand.

In Figure 5A, the radial distribution functions (RDFs) between one of the important cleft amino acids (Ser-79) and the oxygen atoms of water are plotted. From the RDFs, it was observed that the closed (unliganded) form housed a significant number of water molecules in the cleft region. On the other hand, the cleft region was ‘dry’ when the ligand is present. It is understandable that the ligand favorably interacts with both domains and tries to keep them close to one another. In this way, the ligand bound closed form gains its stability. This aspect was well-established in previous studies for similar systems.^13^ However, the unanswered question is how does the closed conformation remain closed even if there is no ligand in between the domains? Interestingly, several water molecules can be found to infiltrate the cleft region and form extended water-mediated bridging hydrogen bonds, as shown with the help of some representative snapshots in Figure 5B–D. In Figure 5B, water molecules can be seen to form a four-membered ring whose four vertices were H-bonded to four different amino acid side chains. Therefore, water molecules were seen to play an active role in the stability of the closed conformation without the presence of the ligand.

**Figure 5:**
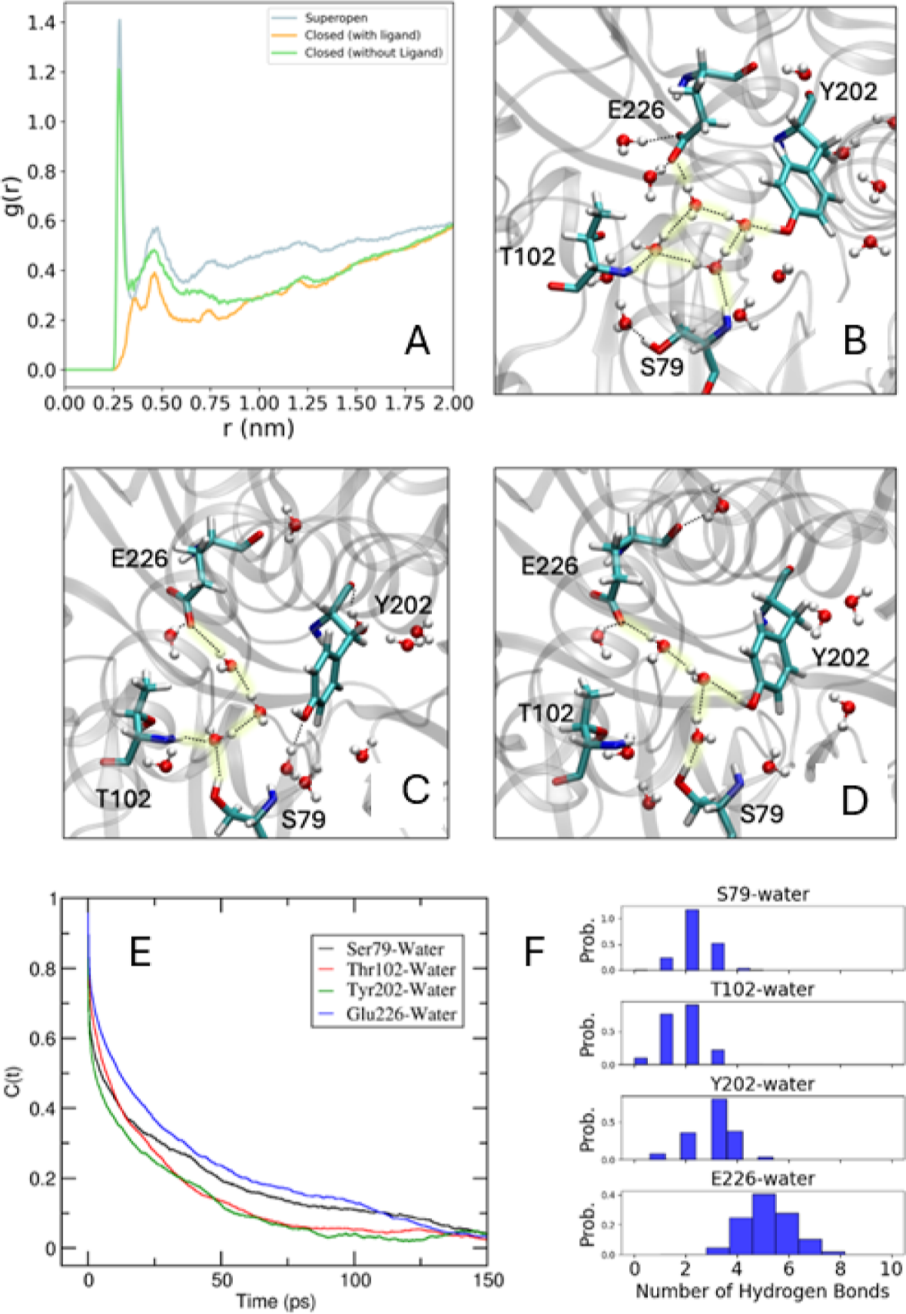
(A) Radial distribution function between S79 and water from unbiased trajectories for the superopen, closed (with ligand), and closed (without ligand) conformations. (B), (C), and (D) are representation of water-mediated bridge hydrogen bonding (highlighted in yellow) between E226, Y202, S79, and T102 in the closed form of the protein without ligand. Such interaction acts as anchors between the two domains of the protein and makes the closed state stable in the absence of the ligand. The H-bonding interaction was observed by using the geometric criteria given by Luzar et al. with *r*_cut_ = 3.5 Å and θ_cut_ = 30°.^43^ (E) Hydrogen bonding time correlation function (HBTCF) of different polar amino acids in the cleft of the LIV-binding protein with water molecules. (F) Distribution of the number of hydrogen bonds formed by the four cleft amino acids with water. The number of hydrogen bonds is calculated between the amino acid residue, including its backbone C=O and N–H, and water molecules.

To quantify the time scales associated with the hydrogen bonds shown in Figure 5B–D, the hydrogen bond time correlation functions (HBTCF) were calculated which can be written in terms of the Heaviside step function, as shown in eq 1:

1 $$C\left(t\right)=\frac{<h\left(0\right)h\left(t\right)>}{<h>}$$

where for a given time point, *h*(*t*) = 1 if a donor–acceptor pair is hydrogen-bonded and *h*(*t*) = 0 otherwise. Here, to define the existence of hydrogen bonds, the geometric criteria proposed by Luzar et al. are used, with *r*_cut_ = 3.5 Å and θ_cut_ = 30°.^43^ To calculate the HBTCF, three independent 500 ps trajectories were run with a 2 fs coordinate saving frequency. The resultant graphs are plotted in Figure 5E. From these graphs, the time scales were obtained with a biexponential fit and written down in Table 1. Figure 5F contains the distribution of the number of hydrogen bonds between different amino acid–water pairs, the average values of which are also given in Table 1. Note that during the HBTCF and *n*_HB_ calculations, the whole amino acid residues were considered including their peptide backbone C=O and N–H. This is because the backbone was also involved in hydrogen bonding with water which plays a major role in holding the two domains in the absence of the ligand [Figure 5B–D].

**Table 1:** Time Scales of the Hydrogen Bonding Time Correlation Function Obtained by Fitting the Traces Shown in Figure 5E with a Biexponential Function^a^

amino acid	*a*_1_	τ_1_ (ps)	*a*_2_	τ_2_ (ps)	<τ> (ps)	<*n*_HB_>
SER 79	0.44	0.26	0.47	34.7	16.4	2.17
THR 102	0.42	1.12	0.57	35.8	21.1	1.64
TYR 202	0.51	0.37	0.49	40.7	20.1	2.65
GLU 226	0.46	2.94	0.60	78.4	48.4	5.18

a The average time scale <τ> is the area under the biexponential decay. The last column contains the time-averaged number of hydrogen bonds for the respective amino acid–water pairs.

From the data, it can be seen that the negatively charged glutamate (residue id 226) contributes the most to both the number and the longevity of hydrogen bonds with water. The other three residues, namely, serine, threonine, and tyrosine, form hydrogen bonds of comparable lifetime with water. In comparison, computational studies on bulk water using different models give a value of <τ> close to only a few ps.^44,45^ However, further understanding of the various contributors in free energy, including solvation entropy, is needed to connect the hydrogen bonding with the stability.

## Conclusion

The conformational fluctuations of the LIV binding protein and its interaction with ligands, with a focus on the dynamics of ligand escape, are studied. Previous hypotheses suggested that the ligand was necessary to sustain the closed conformation of the LIV binding protein. However, the results of this study demonstrated that the closed conformation could remain stable without the ligand, primarily due to the stabilizing effect of water-mediated hydrogen bonds bridging polar side chains of domain-1 and domain-2. On the other hand, the ligand is not sufficient to hold the protein in its closed form as spontaneous domain separation is observed from unbiased MD simulation. These findings challenge the conventional view and provided new insights into the structural behavior of the protein under ligand-free as well as ligand-bound conditions.

Two distinct pathways for ligand escape are identified. In one pathway, the ligand escapes after domain opening, while in the other, ligand escape occurs either simultaneously with or prior to domain opening. A two-dimensional free-energy landscape (2d-FEL) with respect to two collective variables: interdomain distance and protein–ligand distance, unveiled several stable/metastable states during ligand escape. From this 2d-FEL, the dominant pathway is recapitulated to be the one where domain separation occurs first, followed by ligand escape.

Although the kinetics (*k*_on_ and *k*_off_) were experimentally determined for other PBPs, such as the glutamate-binding protein,^46^ they remain unknown for the LIV-binding protein. The free-energy barriers from Figures 4 and 3 could, in principle, be converted to rate constants. Nevertheless, simple rate theories, including transition state theory, may not be applicable here because the barrier is broad and frictional contributions are significant.

While this study characterizes the primary unbinding pathways, future work utilizing more complex collective variables, such as the hydration state of the binding pocket, could further resolve the energetic contribution of water-bridging effects. Furthermore, applying this framework to other PBPs will be essential to determine if the observed sequence of domain opening and ligand release is a conserved mechanism across the protein family. I note that the effect of ions or varying protonation states of the amino acids (and the ligand) on the modulation of the unbinding landscape remains an open question. Future work utilizing constant-pH molecular dynamics could be particularly valuable to determine how fluctuations in the local electrostatic environment of the cleft residues and the ligand itself might shift the relative stability of the escape pathways identified here.

Taken together, the distinct Ile escape pathways, the domain 2 bound intermediates, the peripheral metastable sites, and the water-bridged closed apo state identified here delineate a set of conformational and hydration states that are, in principle, druggable. These mechanistic features can be exploited in future structure-based efforts to design small molecules that block branched chain amino acid transport by stabilizing specific nonproductive intermediates along the escape route or by perturbing the structured water network that supports the closed conformation in the absence of ligand.

## Supplementary Material



## Data Availability

The simulation files and trajectories can be accessed through the Zenodo repository (DOI: 10.5281/zenodo.18249438). All other information is available from the author of this article upon reasonable request.

## Notes

Proof of convergence for the two-variable metadynamics; demonstration of two different ligand escape pathways observed from one-variable metadynamics runs; distributions of interdomain separation distances and interdomain angle for the open, closed (liganded), and closed (unliganded) protein conformations; and spontaneous ligand escape as observed from another independent unbiased trajectory (trajectory-2) (PDF)

## †

Department of Chemistry, Syracuse University, 111 College Place, Syracuse, New York 13244, USA
