# Supplementary material for: Microscopic Insights into a Ligand Escape Pathway and Energetics in Leucine–Isoleucine–Valine Binding Protein
Source: ACS Omega. 2026 Apr 8;11(15):22787–95. doi: 10.1021/acsomega.5c12025 (PMC13103812; doi:10.1021/acsomega.5c12025)
Supplement: Supplementary file 1 [file ao5c12025_si_001.pdf]

Supporting Information for

Microscopic Insights into Ligand Escape Pathway and Energetics in Leucine-Isoleucine-Valine Binding Protein

Jayita Das*

Department of Chemistry, Boston University, Massachusetts, U.S.A.

*E-mail: jdas02@syr.edu

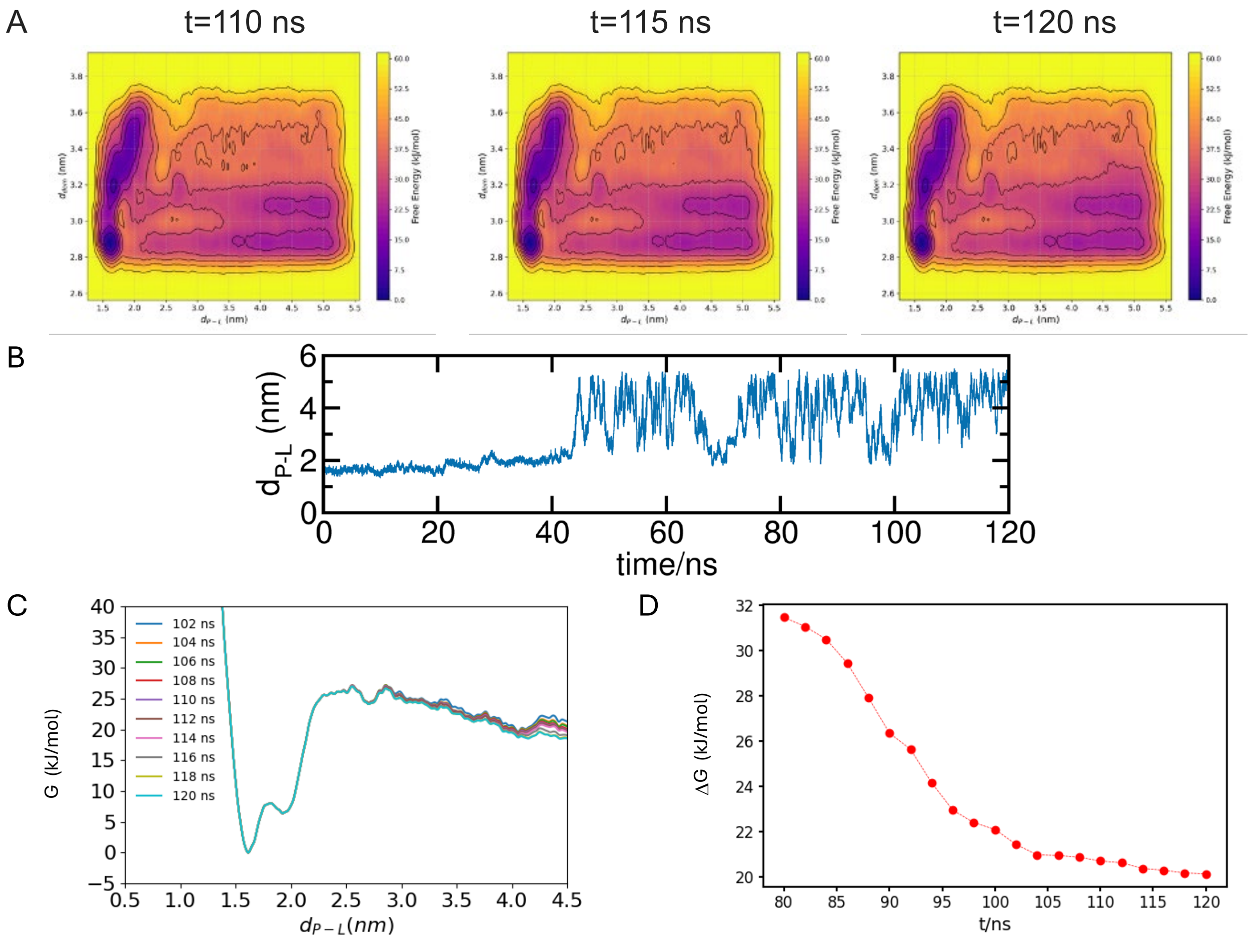

Figure S1: (A) The two-dimensional free-energy landscape (FEL) at three different end points, namely t = 110 ns, 115 ns, and 120 ns. (B) Diffusion of the slower collective variable, $d_{P\text{-}L}$ with respect to metadynamics run time. (C) The one-dimensional projection of the FEL at 10 different end points. (D) The difference of free energy, ΔG between the ligand bound and unbound states ($d_{P\text{-}L}$ ~ 4 nm) with respect to simulation end time. The change of both the one and two dimensional

the free energy landscape with time is negligible. The collective variable is diffusive over the entire range of $d_{P\text{-}L}$ and the variation of ΔG reaches a plateau. These data prove the convergence of the two-variable metadynamics run.

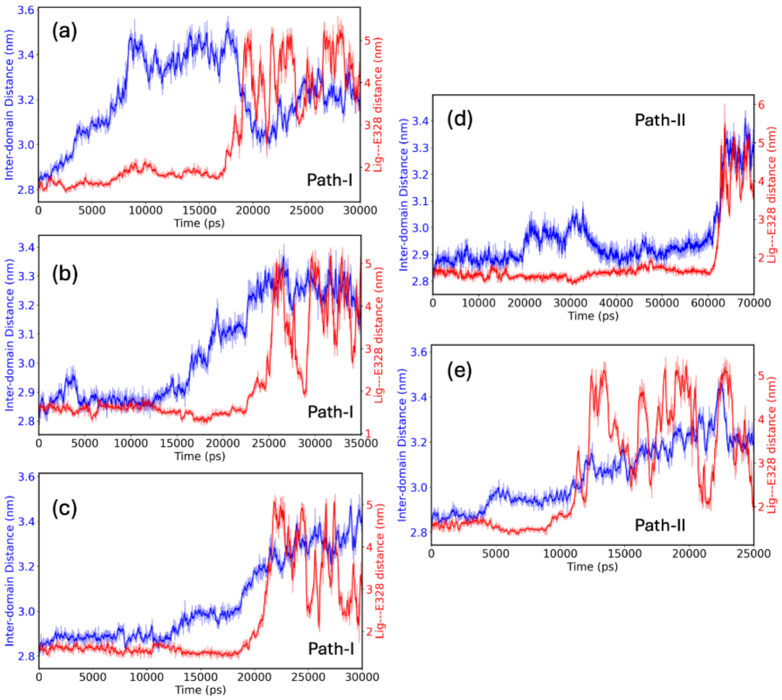

Figure S2. Demonstration of two different ligand escape pathways observed from one-variable meta-dynamics runs. The inter-domain COM distances of the protein (blue) are plotted along with the distances between the protein fulcrum (C_α of E328) and the COM of the ligand (red). Panels (a), (b), and (c) show that domain separation occurs before the ligand has escaped from the cleft. On the other hand, panels (d) and (e) show that either ligand escape and domain separation occur synchronously, or ligand escape happens before the domain separation.

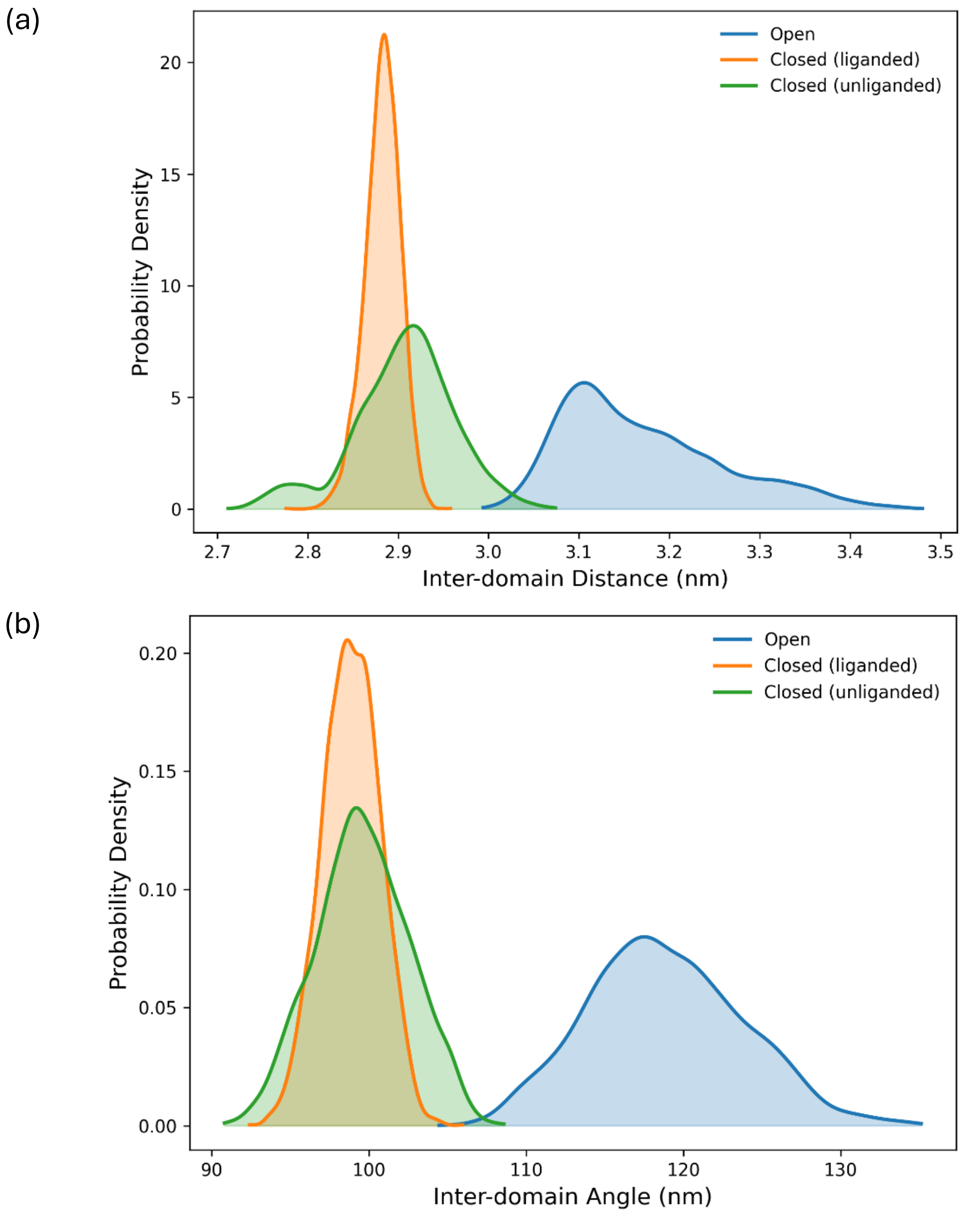

Figure S3. (a) The distributions of Inter-domain separation distances for the open, closed (liganded), and closed (unliganded) along the 100 ns unbiased simulation trajectory. (b) The distribution of Inter-domain angles between the two vectors connecting the fulcrum with the center of mass of each domain as again described in Figure 1B of the main text. These data suggest that the unliganded closed conformation can survive in its closed conformation for ~100 ns as the distributions for the 'true open' conformation (PDB ID 1Z15) is seen to be right sifted with minima overlaps with the rest.

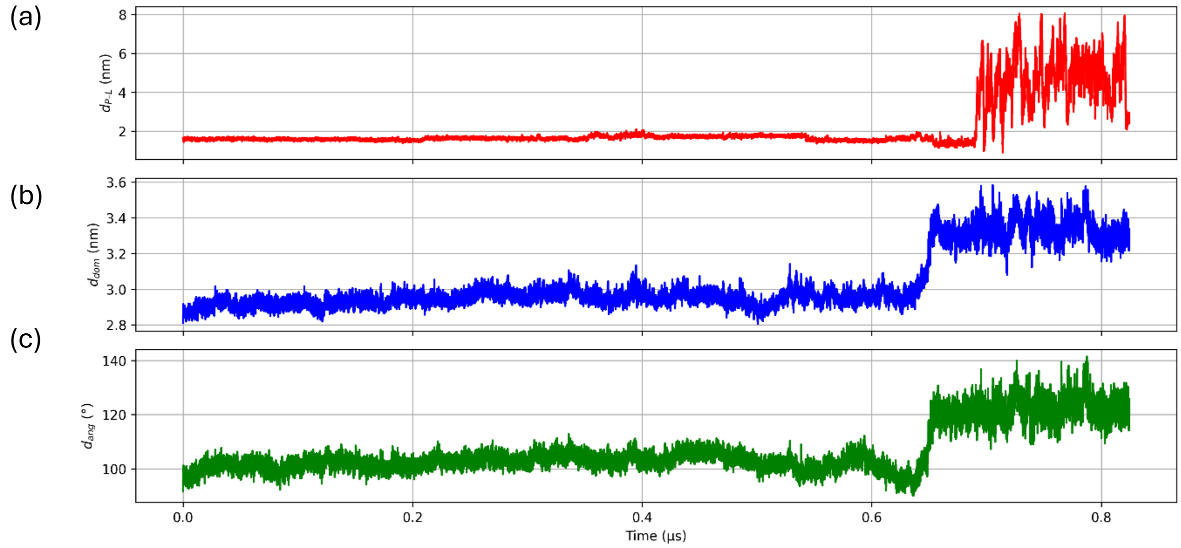

Figure S4. Observations from unbiased 'Trajectory-2': (a) Evolution of the distance between the protein fulcrum and ligand COM with time. This captures the spontaneous ligand escape at around 680 ns. The conformational evolution of the protein: (b) change in the inter-domain distance (d_{dom}) and (c) the inter-domain angle (d_{ang}) with time. This shows the domain separation precedes the ligand escape, as also found from the 'Trajectory-1' reported in the main text.
